# Patterns of Suicidal Ideation and Behavior in Northern Ireland and Associations with Conflict Related Trauma

**DOI:** 10.1371/journal.pone.0091532

**Published:** 2014-03-19

**Authors:** Siobhan O'Neill, Finola Ferry, Sam Murphy, Colette Corry, David Bolton, Barney Devine, Edel Ennis, Brendan Bunting

**Affiliations:** 1 Bamford Centre for Mental Health and Well Being, University of Ulster, Londonderry, Northern Ireland, United Kingdom; 2 Initiative for Conflict Related Trauma, Co. Fermanagh, Northern Ireland, United Kingdom; Univ of Toledo, United States of America

## Abstract

In this study, data from the World Mental Health Survey's Northern Ireland (NI) Study of Health and Stress (NISHS) was used to assess the associations between conflict- and non-conflict–related traumatic events and suicidal behaviour, controlling for age and gender and the effects of mental disorders in NI. DSM mental disorders and suicidal ideation, plans and attempts were assessed using the Composite International Diagnostic Interview (CIDI) in a multi-stage, clustered area probability household sample (N = 4,340, response rate 68.4%). The traumatic event categories were based on event types listed in the PTSD section of the CIDI. Suicidal ideation and attempts were more common in women than men, however, rates of suicide plans were similar for both genders. People with mood, anxiety and substance disorders were significantly more likely than those without to endorse suicidal ideation, plan or attempt. The highest odds ratios for all suicidal behaviors were for people with any mental disorder. However, the odds of seriously considering suicide were significantly higher for people with conflict and non-conflict–related traumatic events compared with people who had not experienced a traumatic event. The odds of having a suicide plan remain significantly higher for people with conflict-related traumatic events compared to those with only non-conflict–related events and no traumatic events. Finally, the odds of suicide attempt were significantly higher for people who have only non-conflict–related traumatic events compared with the other two categories. The results suggest that traumatic events associated with the NI conflict may be associated with suicidal ideation and plans, and this effect appears to be in addition to that explained by the presence of mental disorders. The reduced rates of suicide attempts among people who have had a conflict-related traumatic event may reflect a higher rate of single, fatal suicide attempts in this population.

## Introduction

Suicidal ideation and behaviour are important public health issues, not least because they give us an insight into the factors associated with death by suicide, but also because attempted suicide and non-fatal self-injury represent an important cause of morbidity. When both the direct costs of the suicide and the indirect costs associated with loss of earnings and the unpaid work of the deceased are considered, it is estimated that each suicide in Northern Ireland costs £1.4 million [1] and that the average lifetime costs of each suicide in EU countries is €2 million [2]. The causes of suicide are many, however mental disorders are an important risk factor with up to 90% of those who die by suicide having a mental disorder [3], [4]. Nonetheless, most people with a mental disorder do not go on to die by suicide and many people who die by suicide have no recorded mental health disorder [5] it is therefore important that the associations between suicide and other factors, such as trauma, are examined. International studies of trauma and suicidal behaviour have demonstrated that traumatic events are themselves associated with suicide with the risk of suicidal behaviour increasing with the number of traumatic events endorsed. This association is in a dose response fashion however as a greater number of events are added the strength of the association begins to be reduced [6]. In addition, these studies have shown that certain traumatic event types, those relating to sexual and interpersonal violence are associated with a higher risk of suicidal ideation and behaviour than others [6], [7].

The Northern Ireland (NI) conflict offers an opportunity to examine the associations between trauma and suicidal behavior. Figures highlight an increase in the number of registered suicides in Northern Ireland [1]. This has raised questions regarding the role of the conflict in explaining the increased rates of suicide in the years following the peace agreement in 1998 [8]. Several recent papers based on findings from the World Mental Health Survey Initiative have demonstrated high rates of mental disorders in Northern Ireland [9], [10]. The rates of Post Traumatic Stress Disorder in Northern Ireland is the highest of all the countries involved in the initiative including several countries which have experienced recent civil conflict such as Israel, South Africa and the Lebanon [9]. When the event types are categorized into conflict and non-conflict related trauma (with sexual violence and sudden death of a loved one in the latter category) the prevalence of conflict related trauma in Northern Ireland is 39%. The most frequently reported event in this category was ‘civilian in a place of ongoing terror’ (19.5%) followed by ‘witnessed death or a serious injury’ (16.9%). The risk of mental disorder and PTSD is elevated among those exposed to any conflict related traumatic event compared to those exposed only to non-conflict related traumatic events [11]. The association between experience of the conflict and increased risk of self-harm among adolescents was also demonstrated in the NI Lifestyle and Coping survey [12]. This provides further evidence of the need to examine trauma in the context of suicide risk.

This paper presents the first nationally representative data on the lifetime prevalence and risk factors for suicidal ideation and behavior in Northern Ireland from the Northern Ireland Study of Health and Stress (NISHS), part of the world mental health survey initiative [13]. In order to provide a stringent test of the association between trauma and suicidal behavior, mental disorders were controlled for.

## Methods

Ethics statement: Ethical approval for the study was granted by the University of Ulster ethical committee. Written informed consent was obtained from the participants in this study for their involvement in the research.

The NISHS was the largest epidemiological study of mental health in NI. A multi-stage, clustered, area probability household sample was drawn based on the structure and information from the 2001 NI census. The sample size was 4,340 and the response rate was 68.4%. Data was cleaned and missing data collected or imputed prior to the analysis. See Bunting et al. for further details of the sampling procedures and quality assurance strategies [9]. The NISHS survey instrument was administered in two sections; all participants completed Section 1, section 2 was then administered to respondents who met the criteria for any core disorder, an additional 50% of individuals who were subthreshold core disorder cases, and a 25% sample of all other individuals (*n* = 1,986). This sampling strategy enabled the computation of weights to adjust for differential selection for Section 2. Weights to minimize the effects of bias included information relating to sample selection, nonresponse, and poststratification factors such as age, sex, and geographical region [14]. The NI population characteristics at the midpoint of the data collection period were used in these weight calculations.

The survey instrument was the WMH Composite International Diagnostic Interview (WMH-CIDI) [13]. This is a comprehensive, fully structured interview for the assessment of mental disorders according to the ICD-10 Classification of Mental and Behavioural Disorders: Clinical Descriptions and Diagnostic Guidelines (ICD-10) [15] and DSM-IV criteria [16].

Lifetime suicidal behaviour was assessed using three questions from the suicidality section in part two of the WMH-CIDI: “Have you ever seriously thought about committing suicide?”, “Have you ever made a plan for committing suicide?”, and “Have you ever attempted suicide?”.

Traumatic events were assessed in the PTSD section of part two of the WMH-CIDI. Participants were presented with 28 types of traumatic events and asked whether they had experienced them during their lifetime and if they endorsed a particular event, they were asked the age at which they first experienced this event type. The research team identified events that were presumed to be conflict-related, drawing upon a previous study of conflict in Lebanon [17]. Individuals were assigned to a conflict-related category if they experienced any one of the following events from 1968 onwards: combat experience, peacekeeper in a place of war, unarmed civilian in a place of war, civilian in a place of ongoing terror, refugee, kidnapped, man-made disaster, beaten by someone other than parents or partner, mugged or threatened with a weapon, witnessed someone being killed or seriously injured, purposely caused injury or death, or saw atrocities. The event types classified as non-conflict related included rape and sexual violence, death or illness of a loved one or diagnosis with a life threatening condition. It is also likely that a proportion of unexpected deaths and traumatic events involving loved ones could be associated with the NI conflict, however we did not categorise these event types as conflict-related. This is therefore likely to be a conservative estimation of conflict-related trauma. Mental disorders were assessed on the basis of DSM criteria [16] again using the WMH-CIDI.

Chi squared tests were used to assess whether the difference in proportions between categories were statistically significant. The association between traumatic event types and suicidal ideation, plan and attempt, controlling for the effects of any lifetime mental disorder, was examined using logistic regression. The reference category for the logistic regression was not having endorsed suicidal ideation (“seriously considered suicide”). The analysis incorporated weights to adjust for the differential selection for Section 2, sample selection, nonresponse, and poststratification factors, age, sex, and geographical region [14]. All analyses were implemented using STATA version 10.0 [18].

## Results

Table 1 provides a description of the rates of suicidal ideation, plans and attempts among men and women in Northern Ireland. Women are more likely than men to report having seriously considered suicide (10.6% compared with 7%, p <0.05). Similar proportions of men and women report having made a plan for suicide. Women are also significantly more likely than men to make a suicide attempt (4.3% and 2.3% respectively, p<0.05). The proportion of those with ideation who make a suicide attempt is 41.4% for women and 33.2% for men, whilst 62.0% of women and 38.7% of men who make a suicide plan also make a suicide attempt.

**Table 1:** Prevalence (%) of lifetime suicide ideation, plan, and attempts among women (n = 2441) and men (n = 1899) in the NISHS.

	Female	Unweighted n	CI	Male	Unweighted n	CI
Lifetime suicide ideation	10.6%^1^	276	9.3–12.0	7.0%	159	5.9–8.2
Lifetime suicide plan	2.5%	73	2.0–3.2	2.4%	55	1.8–3.1
Lifetime suicide attempt	4.3%^1^	122	3.6–5.3	2.3%	52	1.7–3.1
Among those w/suicide ideation	41.4%	122	-	33.2%	52	-
Among those w/suicide plan	62.0%	122	-	38.7%	52	-

1 p<0.05

- missing CI and p value due to stratum with single sampling unit.

Table 2 shows the proportions of people with any mood, anxiety or substance disorder and suicidal ideation, plan and attempt. People with these disorders are significantly more likely than those without, to endorse suicidal ideation plan or attempt. The risk for people with mood disorders is 30.2% for ideation, 9.1% for plan and 12% for attempt. For any anxiety disorder the figures are 25.6%, 9% and 8.6% and for substance disorder the rates were 23.4%, 7.9% and 9.3%.

**Table 2:** Proportions endorsing suicidal ideation, plans and attempt among those with each category of mental disorder.

	Any mood disorder (95% CI)	No Mood disorder (95% CI)	Any Anxiety disorder (95% CI)	No Anxiety disorder (95% CI)	Any substance disorder (95% CI)	No substance disorder (95% CI)
**Seriously considered suicide**	30.2%^1^ (26.8–33.7)	3.8% (3.2–4.5)	25.6%^1^ (21.6–30.1)	4.4% (3.5–5.7)	23.4%^1^ (18.2–29.6)	7.0% (5.9–8.3)
**Suicide plan**	9.1%^1^ (7.2–11.3)	0.9% (0.7–1.3)	9.0%^1^ (6.3–12.7)	0.8% (0.5–1.4)	7.9%^1^ (4.6–13.2)	1.9% (1.3–2.6)
**Suicide attempt**	12%^1^ (9.8–14.5)	1.4% (1.0–1.8)	8.6%^1^ (6.5–11.2)	1.4% (0.9–2.2)	9.3%^1^ (6.5–13.0)	2% (1.5–2.8)

1 χ^2^ test indicates a significant difference compared to those with no disorder p = 0.0001.

Table 3 presents the association between the experience of any lifetime trauma and lifetime suicidal behavior and demonstrates that individuals who have experienced any traumatic event have a significantly elevated risk of having seriously considered suicide, having planned suicide and having attempted suicide. There were also significant differences across the three trauma categories in terms of suicide ideation and plans, with people who had a conflict related traumatic event being more likely to have suicidal ideation and plans. However people who had experienced conflict related traumatic events were less likely than those who endorsed other traumatic event types to have attempted suicide.

**Table 3:** Proportions endorsing suicidal ideation, plans and attempt among those with no traumatic event, only non-conflict related traumatic events, conflict-related traumatic events and any traumatic event.

	No traumatic event (95% CI)	Only non-conflict related events (95% CI)	Conflict related traumatic events (95% CI)	Any traumatic event (95% CI)
**Seriously considered suicide**	3.8% (8.7–5.4)	10.5^1^ (8.0–13.6)	14.2%^1^ (11.6–17.2)	12.9%^1^ (10.9–15.0)
**Suicide plan**	1.1% (0.6–2.0)	2.4%^3^ (1.4–4.3)	4.5%^4^ (3.0–6.8)	3.8%^2^ (2.7–5.3)
**Suicide attempt**	1.2% (0.6–2.3)	5%^5^ (3.4–7.3)	3.8%^5^ (2.8–5.3)	4.3%^3^ (3.3–5.5)

χ^2^ test indicates a significant difference compared to those with no traumatic event ^1^P = 0.0000 ^2^p = 0.0002 ^3^p = 0.0001 ^4^p = 0.0018 ^5^p = 0.0024.

Three multivariate logistic regressions were undertaken assessing the relative odds of each of the three suicidal behaviour categories for people with any mental disorders (with no mental disorder as the reference category), conflict and non-conflict related traumatic events (using no traumatic events as the reference category, sex (with male as the reference category) and age group (with 18–34 years as the reference category). The results are shown on table 4. The highest odds ratios for all suicidal behaviours are for people with any mental disorder. However, the odds of seriously considering suicide remain significantly higher for people with conflict and non-conflict-related traumatic events compared with people who have not experienced a traumatic event. The odds of having a suicide plan remain significantly higher for people with conflict related traumatic events compared with those who have only non-conflict related events and no traumatic events. Finally, the odds of suicide attempt are significantly higher for people who have only non-conflict related traumatic events compared with conflict related events and no traumatic events.

**Table 4:** Logistic regression analyses of socio-demographic and conflict related trauma correlates of suicidal ideation, plans and attempt.

	Seriously considered suicide OR (95% CI)	Suicide plan OR (95% CI)	Suicide attempt OR (95% CI)
**Any mental disorder**	8.6 (5.6–133)^1^	15.8 (6.2–40.7)^1^	15.2 (6.7–34.1)^1^
**Only non-conflict related trauma**	1.8 (11–2.9)^2^	1.5 (0.6–3.6)	2.6 (1.1–5.6)^4^
**Conflict related trauma**	23 (1.5–3.6)^1^	2.2 (1.0–4.8)^3^	1.8 (0.9–3.9)
**Sex (female)**	1.3 (0.9–1.8)	0.8 (0.4–1.4)	1.6 (1.0–2.8)
**Age 35**–**49**	1.1 (0.7–1.7)	1.8 (0.8–3.9)	1.2 (0.6–2.2)
**Age 50**–**64**	1.1 (0.7–1.8)	1.6 (0.6–4.2)	1.0 (0.5–1.9)
**Age 65+**	0.7 (0.4–1.4)	0.6 (0.2–2.3)	0.6 (0.2–1.5)

1 p = 0.000 ^2^p = 0.024 ^3^p = 0.05 ^4^p = 0.023

For sex the comparator category is male so only the OR for females is shown.

For age the comparator category is 18-34.

## Discussion

This is the first study to examine suicidal ideations and behavior in Northern Ireland and the associations with mental disorders and conflict related trauma. In keeping with the extant literature from Western cultures [19], suicidal ideation is more common among females in all age groups (except for those aged over 65). There are similar rates of plans and attempts for men and women. The elevated rates of suicide attempts among ideators reinforces the need for the assessment of suicidal ideation among vulnerable individuals and the need to treat ideation seriously as an indicator of suicide risk. In addition, the proportion of unplanned attempts emphasizes the need for further investigation of the phenomenon of impulsive suicides [20].

Risk of suicidal ideation, plans and attempts are significantly higher for individuals with all categories of mental disorders. In the multivariate model, mental disorder remains the strongest predictor of all types of suicidal behavior. This is in keeping with the patterns identified in other World Mental Health Survey countries [4]. As expected, having experienced any traumatic event is associated with an increased risk of suicidal thoughts, plans and attempts. This supports the findings from international studies linking trauma with suicidal behavior [6], [7], [21], [22]. Experiencing a conflict related traumatic event was associated with a higher rate of suicidal ideation and plans than having experienced a non-conflict related traumatic event or having not experienced a traumatic event. However, in the multivariate model, which also considers the impact of having any mental disorder, as well as age and gender, having only a non-conflict related traumatic event is associated with an increase in risk of attempt, but not plan. Finally, in the multivariate model, having a conflict related traumatic event is associated with an increase in risk of plan, but not attempt. The multivariate model therefore illustrates that the risk of suicide ideation associated with all types of traumatic events is additional to that of mental disorder. The risk of suicide plan associated with having a conflict related traumatic event is also additional to the risk associated with having a mental disorder. The same pattern holds for having a non-conflict related traumatic event in relation to suicide attempt.

The decrease in odds ratios when mental disorders are included in the model demonstrate that some of the effect of traumatic events on suicidal behavior is through the associations with mental disorders. This is not surprising given the associations between conflict related trauma and DSM mental disorders in Northern Ireland. There is also evidence that in this population trauma related conflict is associated with more severe and enduring mental disorders [10] which are themselves a predictor of suicidal behavior [6]. The additional risk of ideation, and attempts is again not surprising. A cross national analysis of data from several world mental health countries indicated that interpersonal and sexual violence are associated with the highest risk of suicide ideation, plans and attempts [6]. In this analysis we categorized sexual violence as being not related to the conflict.

This study builds on previous evidence illustrating the high prevalence of conflict-related traumatic events in the Northern Ireland population. It is estimated that 60.6% of the population have experienced a traumatic event, 39% have experienced a conflict-related traumatic event and 16.9% witnessed death or serious injury [11] This study is the first to show that people who reported a conflict related traumatic event were more likely than those with other traumas and no traumas to have seriously considered suicide and made a suicide plan, even when we control for the effects of mental disorders. But they were less likely to endorse a suicide attempt in both the bivariate and multivariate models. The other world mental health countries did not categorise event types in the manner that we did, however in these countries the broad ‘exposure to war’ category did carry a higher risk of suicide attempt than other types of traumatic events [4], [6]. We have only found one other study examining suicidal behavior and the NI conflict and this was a study of self harm among adolescents. In this study both boys and girls were significantly more likely to report engaging in self harm if they had experienced ‘troubles’ related events (being involved in an explosion or riot, being a victim of violence or having a relative, friend or someone known to them killed or injured [12]).

The results are based on a small sample of suicidal individuals in the NI population and as such must be interpreted with caution. There are a variety of explanations for these findings which need to be tested in subsequent research. It may be that people exposed to conflict related traumatic events are also exposed to other factors which protect against suicide attempts. Tomlinson [8] argued that, in keeping with Durkhiem's theories of suicide [23], the strong social networks which characterized Northern Ireland during the conflict protected against suicide. However this contradicts the finding that people with a conflict related traumatic event have higher rates of suicidal ideation and suicide plans. An alternative explanation is that those who are exposed to conflict related traumatic events are more likely to die following their first suicide attempt and are therefore missing from the NI Study of Health and Stress. In NI, men were more likely than women to have experienced a conflict related traumatic event [11]. It is widely recognized that women are more likely to make a suicide attempt than men. However men are more likely than women to make a single fatal suicide attempt [19]. They also choose more lethal methods generally and are more likely to use firearms as the means of suicide [24]. Finally, there is evidence suggesting that people are more likely to make a fatal suicide attempt or choose a more lethal means if they have been habituated (following repeated exposure) to pain, violence and/or death [5], [25]. The differential relationship between suicidal ideation and plans versus attempts and conflict related trauma also supports recent theories of suicidality (for example the Integrated Motivational volitional model) [26] which argue that the factors which are associated with suicidal ideation are different from those which determine whether thoughts are acted upon. More work is required to provide a better understanding of the volitional factors which increase the likelihood that suicide ideation is translated into a suicide attempt. Together, these theories suggest that those with a conflict related traumatic experience are more likely to die following their first suicide attempt. However, it is only through the collection and analysis of valid and detailed data on deaths by suicide in NI that this hypothesis may be tested.

The results should be interpreted in the light of the study's limitations. The study relied on retrospective recall and reporting of traumatic events. Suicidal behavior is also a sensitive and stigmatized subject and responses to questions on both may be subject to recall and reporting bias. Whilst weights were used to account for the variation in response rates among subpopulations there may remain an element of non-response bias. In addition individuals who endorsed items from both the conflict and non-conflict related trauma categories were categorised as having a conflict related traumatic event. Therefore this category is more likely to include individuals with cumulative traumas, which are also associated with suicidal behavior [6].

These findings add further weight to the evidence for the routine assessment of suicidal ideation in high risk populations, particularly those with mental disorders. They demonstrate an increased risk of adverse mental health outcomes among people who have experienced traumatic events relating to the NI conflict. Those who have suffered as a consequence of the conflict should therefore not only be monitored and provided with evidence based treatments for the mental disorders, but also for suicidal thoughts plans and behaviors.
